# Supplementary material for: Haplotag: Software for Haplotype-Based Genotyping-by-Sequencing Analysis
Source: G3 (Bethesda). 2016 Jan 26;6(4):857–63. doi: 10.1534/g3.115.024596 (PMC4825656; doi:10.1534/g3.115.024596)
Supplement: Supporting Information [file supp_6_4_857__index.html]

Haplotag: Software for Haplotype-Based Genotyping-by-Sequencing Analysis — Supporting Information 

# Haplotag: Software for Haplotype-Based Genotyping-by-Sequencing Analysis

## Supporting Information for Tinker, Bekele, and Hattori, 2016

**Files in this Data Supplement:**

- File S1 - Haplotag: Software for Haplotype-Based Genotyping-by-Sequencing (GBS) Analysis User Manual (2016-January-12). (.pdf, 776 KB)
- File S2 - Details of model selection for: HC1. (.zip, 2 KB)
- File S3 - This report shows legacy loci from other work including Huang et al 2014 that match consensus sequences in the current Haplotag production model. (.xlsx, 1.50 KB)
