# Supplementary material for: Haplotag: Software for Haplotype-Based Genotyping-by-Sequencing Analysis
Source: G3 (Bethesda). 2016 Jan 26;6(4):857–63. doi: 10.1534/g3.115.024596 (PMC4825656; doi:10.1534/g3.115.024596)
Supplement: Supporting Information [file supp_g3.115.024596_FileS1.pdf]

File S1

Haplotag:

Software for Haplotype-Based Genotyping-by-Sequencing (GBS) Analysis
User Manual (2016-January-12)

Author: Nick Tinker (nick.tinker@agr.gc.ca)

Citing Haplotag: Tinker, N.A., W.A. Bekele, J. Hattori. 2016. Haplotag: software for haplotype-based genotyping-by-sequencing analysis. G3: genes - genomes - genetics. (in press; please update on publication)

What is Haplotag?

Haplotag is a software program to analyse data from genotyping-by-sequencing experiments in natural and/or experimental populations of biological organisms. It was initially developed to solve specific issues related to the discovery and analysis of genetic polymorphisms in oat: a self-pollinating allohexaploid plant species with no reference genome. While this was the driver for development, Haplotype may find use in other species and other genetic systems.

The absence of a reference genome requires that polymorphisms be discovered from sequence alignments that may span multiple paralogous loci. To identify which polymorphisms are allelic (belonging to a single diploid locus) one needs to test models of polymorphism assortment in genetic populations. A model with two alleles that appear to be homozygous in most inbred lines would be accepted, while a model that implied a large proportion of heterozygotes would be rejected.

Rather than test models based on single SNP loci, Haplotag takes advantage of the fact that short sequence reads from genomic data will often contain multiple SNPs, and that these SNPs form haplotypes that segregate as though they are multiallelic loci. Thus, Haplotag is able to test and fit models with three or more haplotypes that effectively account for all of the SNPs within those haplotypes. Some potential advantages of this include: the ability to discover multiallelic SNPs and high-density SNPs that form complex alignments, the ability to directly quantify the frequency of each multiallelic haplotype, and the ability to use haplotype data in genetic association analysis where it may provide increased statistical power for the discovery of QTLs in phase with a specific haplotype. More discussion of these features will be provided in accompanying publication (provide reference here when published).

In addition to these advantages, Haplotag is designed to produce a highly visual and intuitive “passport view” of each haplotype model that is identified. We believe that the visualization of haplotypes and their assortment in genetic populations can provide useful insight into genetic systems. They are also educational, as they can be used to illustrate the principles of GBS analysis and genetic segregation to students and lay persons. These passports are produced in addition to standard genotype output for every Haplotag analysis. Each passport is a simple HTML file that can be viewed in any web browser. Passports are indexed to a master page that can be searched for a locus of interest. These static HTML pages can be used locally or they can be uploaded to a website in order to provide access to a community of collaborators.

To illustrate this, Figure 1 is a direct, un-modified screenshot of a Haplotag passport. It shows an initial alignment of sequences from which models for two independent loci were selected.

Oat GBS passport file for tag cluster: avgbs_cluster_104

Includes Legacy GBS loci with SNP positions: ***avgbs_68284 (35-A/G) avgbs_6K_63799 (52-C/T) avgbs_204295 (48-C/T)***

Cluster Consensus: TGCAGCTCANTATGATCTCTAGACTAGCCCCACANACCAACACTAGTNTTTNCTGCGCAATTTG

```
             TGCAGCTCANTATGATCTCTAGACTAGCCCCACANACCAACACTAGTNTTTNCTGCGCAATTTG
TagID  Count          1         2         3         4         5         6
570    300   TGCAGCTCAATATGATCTCTAGACTAGCCCCACAAACCAACACTAGTCTTTTCTGCGCAATTTG
571    1261  TGCAGCTCAATATGATCTCTAGACTAGCCCCACAGACCAACACTAGTCTTTTCTGCGCAATTTG
572    320   TGCAGCTCAATATGATCTCTAGACTAGCCCCACAGACCAACACTAGTTTTTTCTGCGCAATTTG
573    1349  TGCAGCTCATTATGATCTCTAGACTAGCCCCACAGACCAACACTAGTCTTTCCTGCGCAATTTG
574    332   TGCAGCTCATTATGATCTCTAGACTAGCCCCACAGACCAACACTAGTCTTTTCTGCGCAATTTG
```

(For details of model selection click HERE)

Best model #1 = 7 - fits 1859 genotypes, with 1% heterozygotes.
Consensus: TGCAGCTCAATATGATCTCTAGACTAGCCCCACARACCAACACTAGTYTTTTCTGCGCAATTTG

```
Locus 1      TGCAGCTCAATATGATCTCTAGACTAGCCCCACARACCAACACTAGTYTTTTCTGCGCAATTTG
TagID  Count          1         2         3         4         5         6
570    300   TGCAGCTCAATATGATCTCTAGACTAGCCCCACAAACCAACACTAGTCTTTTCTGCGCAATTTG
571    1261  TGCAGCTCAATATGATCTCTAGACTAGCCCCACAGACCAACACTAGTCTTTTCTGCGCAATTTG *
572    320   TGCAGCTCAATATGATCTCTAGACTAGCCCCACAGACCAACACTAGTTTTTTCTGCGCAATTTG
```

Best model #2 = 26 - fits 1660 genotypes, with 1% heterozygotes.
Consensus: TGCAGCTCATTATGATCTCTAGACTAGCCCCACAGACCAACACTAGTCTTTYCTGCGCAATTTG

```
Locus 2      TGCAGCTCATTATGATCTCTAGACTAGCCCCACAGACCAACACTAGTCTTTYCTGCGCAATTTG
TagID  Count          1         2         3         4         5         6
573    1349  TGCAGCTCATTATGATCTCTAGACTAGCCCCACAGACCAACACTAGTCTTTCCTGCGCAATTTG
574    332   TGCAGCTCATTATGATCTCTAGACTAGCCCCACAGACCAACACTAGTCTTTTCTGCGCAATTTG
```

Literal segregation of tag presence in test population

Best selected models are on the left -------- haplotypes excuded from the selected locus model(s) are on the right

TaxaID	Project	TaxaName	Locus Model and haplotype IDs				
			Locus-1			Locus-2	
			570	571	572	573	574
T11	CORE	Aarre	0	0	0	0	0
T910	CORE	AC Marie	0	3	0	2	0
T959	CORE	AC Morgan	1	0	0	2	0
T1432	CORE	AC Pinnacle	0	1	0	1	0
T1544	CORE	AC Rigodon	0	0	0	2	0
T1548	CORE	AC Ronald	0	1	0	2	0
T12	CORE	Adrew	0	0	1	0	0
T13	CORE	Ajax	0	0	0	4	0
T14	CORE	Ajay	0	2	0	0	0
T15	CORE	Akiyutaka	0	0	2	0	0
T16	CORE	Allen	0	13	0	0	5
T18	CORE	ARDENTE	8	0	0	2	0
T20	CORE	Assiniboia/S42	0	0	0	4	0
T21	CORE	Aurora	0	0	0	1	0
T22	CORE	Ave117.2	0	1	0	0	0
T23	CORE	Aveny	9	0	0	5	0
T24	CORE	Balado	0	5	0	1	0
T28	CORE	Barra	0	0	1	1	0
T29	CORE	Belinda	0	0	0	4	0
T30	CORE	Bia	5	0	0	1	0

Figure 1. Screenshot of an HTML passport produced by Haplotag in an oat GBS data set. At the top is the initial tag alignment. Below this are models for two independent loci based on mutually exclusive haplotypes from the initial alignment. Haplotypes are identified by "TagIDs" 570 through 574. The lower table shows the literal segregation of these haplotypes in the first 20 taxa (scrolling down would

show a much larger set, in this case approximately 2600 taxa). Numbers in the cells are the actual counts of each tag, which have been shaded to indicate haplotype presence and locus membership.

> *"The easiest way to get started with Haplotag is to unpack the distribution archive, then look for three demo directories named according to the pipelines described in Table 4 and illustrated in Figure 2. Each directory contains a fully functional Haplotag script that works with a set of small annotated demo files.*

Hardware and software requirements:

The current implementation of Haplotag requires a 64-bit Windows operating system. It has been tested on Windows 7, 8, and 10 as well as server versions. The amount of resources depend on the size of project, the size of the genome, the number of taxa, etc. We have been able to perform moderately large analyses on a PC with 8GB of RAM, 4 CPUs, and 200GB of hard disk space, while a project with 3000 taxa in hexaploid oat required 24GB of RAM. Most program steps can take advantage of multiple processors, and speed will scale almost directly with the number of processors, thus a PC with 24 processors could accomplish some jobs 12 times faster than a PC with 2 CPUs. Since Haplotag reads very large input files and writes a large number of small files as output, it will run very slowly when reading and writing across network drives, and we highly recommend running it with all files on local hard disks.

Haplotag is written in Free Pascal using the Lazarus IDE. Both are based on open source community projects available for popular computer including Windows, Linux, and Mac-OS. However Haplotag has so far been compiled and tested for the Windows environment, and users of other systems may need to request the source code and make minor modifications suitable to other environments.

Haplotag does not depend on any other software to run, but it currently requires input files produced by the UNEAK pipeline of the TASSEL software (http://www.maizegenetics.net/#!tassel/c17q9). Our reason for this dependency is that UNEAK is very efficient at parsing raw FastQ files, and separating them into tag count files for each taxa based on multiplex barcodes, and we saw no reason to try and duplicate this function. Learning to use UNEAK may some investment of time, but it will also give you access to an alternative SNP calling pipeline for cross validation of results. Haplotag should call most of the same SNPs as UNEAK, as well as additional SNPs belonging to haplotypes with multiple SNPs and haplotypes from paralogous series which UNEAK will discard. A short helper-tutorial on using UNEAK can be found at the end of this manual.

Haplotag contains an experimental feature to call genotypes directly from raw FASTQ files when running in production mode, given a predefined set of loci from previous projects. This mode is not currently supported, but in future we hope to replace the functionality UNEAK within Haplotag routines.

Installing and Running Haplotag:

Haplotype is contained in a single file "Haplotag.exe" and no installation is required. You should only download Haplotag from a reliable source, preferably directly from the author's distribution site:

http://haplotag.aowc.ca/

Haplotag is executed from a single command followed by the name of an input file. E.g.:

C:\myhaplotagdirectory\haplotag HTinputfile.txt

The input parameter/script file (described below) provides all the information that Haplotag requires to run, including parameter settings and pipeline steps. For those not familiar with using Windows command line, there are four ways to do this:

Option 1) Just double click on "Haplotag.exe" but make sure it is in the same directory as the input file, and the input file must be called "HTinput.txt". For legacy reasons, the name "inputfile.txt" will also work if the default name is not present.

Option 2) Open a command prompt from the Windows start menu and type the command.

Option 3) Write a batch file by typing this command into a text file and saving the file with the extension ".bat". Then click on the batch file to run the program. This option is useful if you wish to walk away and leave your computer to perform multiple analyses (just type multiple commands, one per line).

Option 4) Create a shortcut by right-clicking in a windows folder, selecting "new", then "shortcut". Select "Haplotag.exe" as the target. You may need to follow a wizard to do this in windows 8, then afterward (or directly) edit the properties of the shortcut to look like the form below (directory names are suggestions).

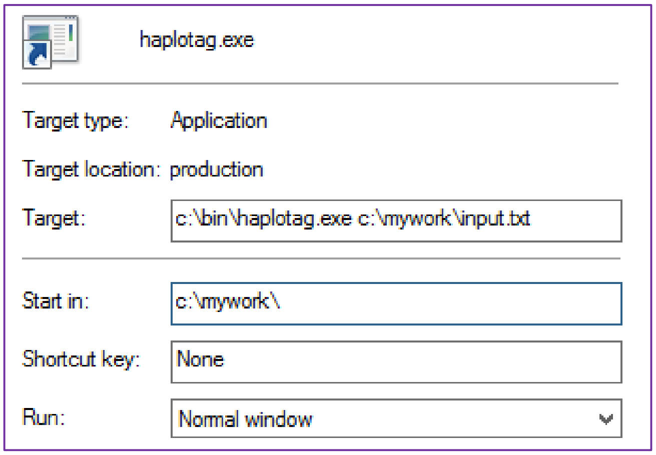

Input Parameter/Script File (default name "HTinput.txt")

The input parameter/script file is a special text file that tells Haplotag exactly what to do and what parameters to use while doing it. You can write or edit this file using Windows Notepad[1]. We

[1] As an alternative to Notepad, we recommend a free third party text editor called Notepad++. This editor is will allow you to view and edit very large text files that Haplotag (and other genomics software) may produce.

recommend that you create and save a new input file for each of your projects. This will provide an audit trail for use in your methods section when you publish your results.
The format of the input file is very simple, as shown below. **Program parameters** come first, and begin with a special word preceded by the strudel (@) and followed by the parameter setting. After this come the **pipeline steps**, each preceded by an exclamation mark (!) and followed (usually) by a single file name. Any line that does not begin with "!" or "@" is ignored as a comment. The sample input files distributed with Haplotag in the demo directory give more extensive examples with additional comments.

```
# Program parameters:
@ProjectName            MyProject
@RSite                  PstI-MspI
# Pipeline steps:
!ReadTaxaIDFile         HTtaxa.txt
!ClusterMergedAll       MergedAll.txt
```

Program parameters

Program parameters should be specified at the beginning of an input file. In some cases it may be possible to change parameters in between pipeline steps, e.g. to change a default path, but this is not recommended or necessary. Program parameters begin with the "@" symbol, and are followed by a single setting. All valid program parameters are shown in Table 1. All parameters have a default value that will be used if the parameter is not set by the user. Since we cannot guarantee the defaults will remain the same for all versions, **we highly recommend that you specify ALL parameters** in your input file. Parameters that are not relevant to certain pipeline steps will be ignored. You may use the defaults shown below as guidance, but please consider that appropriate parameters for GBS analysis are highly dependent on the organism, the technical methods, and the genetic system. Only you, the investigator, can decide what is appropriate, and this may require trial and error.

Table 1. Description of all parameters that can be set in Haplotag input file.

Parameter name	Description	Values	Default setting[2]
@PATH	Default location for input and output files, such as "c:\mydata\haplotag\" (no quotes). ".\" = current directory.	Legal, existing path on your computer.	.\
@ProjectName	Short name for your project. This will appear in your output files.	Any character string, no spaces	Haplotag_project
@ClusterPrefix	Prefix for naming new clusters and loci in discovery modes.	Any character string, no spaces	HC
@MaxBaseDif	This specifies the maximum number of nucleotide differences between two tags before they are joined into the same cluster.		3
@Verbose	Causes Haplotag to report details of model selection in an html file for each	true or false	true

[2] Do not take default settings for granted as they may change in future versions.

	cluster. This will use more disk space and run more slowly.		
@ThreePlus	Writes an HTML index file where only the models with >2 haplotypes at a locus are reported. This makes a shorter index (long indexes load slowly in a browser).	true or false	false
@MaxThreads	Maximum number of threads (computer cores) to use. Setting this to 999 will use every core on your computer and could slow things down a bit. Setting 99 will use all cores except 1.	Any integer, or 99, or 999	99
@MinTagCount	Minimum tag count (across all taxa) for including a tag in a model.	Any integer	10
@MinPres	Minimum frequency of taxa with a given haplotype for that haplotype to be included in a locus model.	Any decimal between 0 and 1	0.002
@MaxPres	Maximum frequency of taxa with a given haplotype for that haplotype to be included in a locus model.	Any decimal between 0 and 1	0.998
@MaxQ	Maximum number of unique haplotypes (will stop reading a file if this is reached).	Any integer	300000000
@MaxS	Maximum number of tag clusters to consider when building clusters.	Any integer	100000000
@MaxTagsToTest	Maximum number of tags in a cluster alignment. Alignments with more tags than this will be discarded. Since Haplotag tests every possible combination of tags, numbers larger than 9 may run slowly. Only relevant in discovery modes.	Any integer	9
@RSite	The restriction enzyme(s) used to make the GBS complexity reduction. Only relevant when reading raw FastQ files. Other combinations may be added later.	PstI-MspI<br>ApeKI	PstI-MspI
@ThreshGeno	The minimum frequency of complete genotypes (relative to the full taxa list) for a model to be selected. Only relevant in discovery modes.	Any decimal between 0 and 1	0.50
@ThreshHet	The maximum overall heterozygote frequency (heterozygotes/genotypes) tolerated in a haplotype model. Only relevant in discovery modes.	Any decimal between 0 and 1	0.10
@ThreshMAHet	The maximum heterozygote frequency tolerated, relative to a single haplotype. This eliminates rare haplotypes that participate in too many heterozygotes. This parameter is not useful in a bi-parental population and can be set to 1. Only relevant in discovery modes.	Any decimal between 0 and 1	0.25
@ThreshTrihet	The maximum frequency of genotypes that contain three haplotypes. Only relevant in discovery modes.	Any decimal between 0 and 1	0
@ThreshMultiHet	The maximum frequency of genotypes	Any decimal between	0

	that contain four or more haplotypes. Only relevant in discovery modes.	0 and 1	
@SkipBC1	This is used only when reading raw FastQ files. It determines whether the first barcode base is ignored when assigning sequence reads to taxa. Because the first sequenced base is often N, this may find additional sequences. Please check that all barcodes are unique after the first base.	true or false	false
@HetRatio	Specifies a threshold ratio of tag count for a minor allele, below which the allele will be dropped. For example if A:B = 1:19 and the HetRatio is 0.06 then the A haplotype will be counted as absent because its ratio is 0.5. When a non-zero tag is counted as absent, it is shown in grey in the passport.	0 and 1	zero

Pipeline steps

The next section of the input file describes the actual pipeline steps that Haplotag will perform. These commands begin with "!", and are described in Table 2. Various commands from this table can be combined into a set of standard pipeline scripts, as illustrated in Figure 2, Table 4, and described later under the section "**Standard Pipelines**". In most cases the command is followed by either a single file name or, if multiple files are required, by a single directory path. You should generally use the default filenames shown, but you may choose to use alternate file names if you have renamed output from previous steps. You may also enter a path as part of these filenames. When Haplotag detects that a filename already contains a path (indicated by the slash "\") then it will not add your default path to the filename. Otherwise it always adds the default path. This is useful if you want to read data from a different directory. This feature is demonstrated in the sample input files.

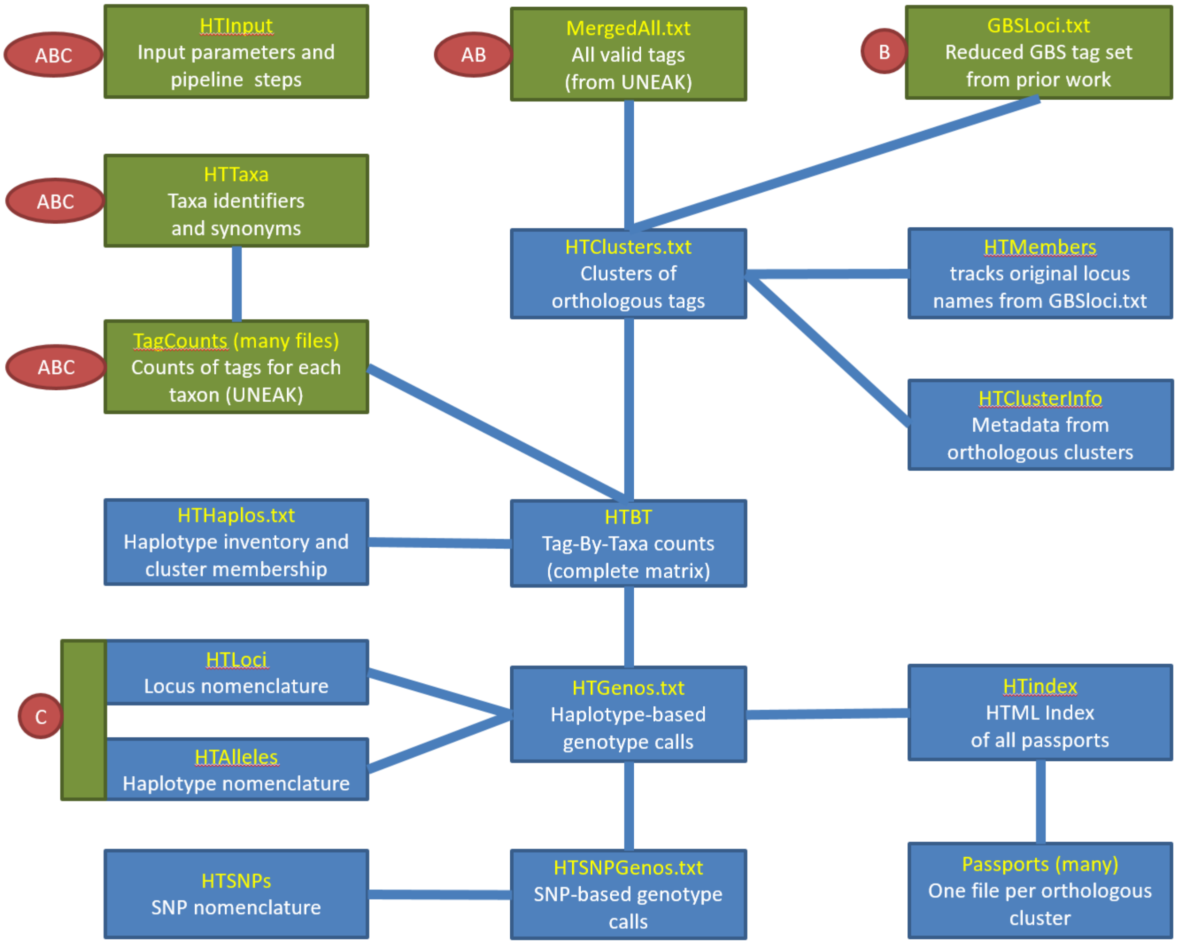

Figure 1. Flow chart showing input files (green), output files (blue) and dependencies (connecting lines) associated with 'Haplotag' GBS discovery software. Default file names are shown in yellow, and are normally appended by ".txt" in the Windows file system. Three alternative pipelines (A, B, and C) are available, with required input labeled for each. The cluster discovery pipeline (A) and the haplotype discovery pipeline (B) start by clustering a complete inventory of tags (A) or a reduced inventory of tags from prior work (B) to produce clusters. In (B), the complete inventory is then aligned against this template to increase the sampling of new haplotypes. A complete tag-by-taxa matrix of tag counts (HTBT) is then formed for all tags belonging to clusters of two or more tags. Other output files are then created based on haplotype model fitting. In the production pipeline, only the files labelled by (C) are required, since genotyping is based on counting copies of haplotype-tags in the output files from previous discovery work.

Table 2. **Description of pipeline commands** that can be combined into a Haplotag script. See **Table 4** for standard pipelines that use these commands in a meaningful sequence of steps. Where indicated, procedures are multithreaded to speed up execution.

Command	Files/data required (see Table 3)	Files/data produced (see Table 3)	Comments

!ReadTaxaIDFile	HTTaxa.txt	(none)	Causes Haplotag to read a list of taxa, including short identifiers, longer entry names, and project membership. This taxa file is required by all Haplotag procedures, and should be read as the first step in every pipeline.
!ReadTaxaKey	HTKey.txt	(none)	Causes Haplotag to read a list of taxa with accompanying barcodes and raw data file names. This is only required when reading raw data for a production run.
!ClusterMergedAll<br><br>*(multithreaded)*	MergedAll.txt	HTClusters.txt<br>HTHaplos.txt<br>HTClusterinfo.txt<br>HTSingletons.txt	Causes Haplotag to read a file containing global tag counts from a project. The MergedAll file is produced by the UNEAK pipeline. After reading the file, Haplotag tries to combine the tags into clusters based on current parameters. Tags that remain as singletons will be written to a separate file that is not normally used. Members of each cluster are written to "HTHaplos".
!ClusterGBSLoci<br><br>*(multithreaded)*	GBSLoci.txt	HTClusters.txt<br>HTClusterinfo.txt<br>HTMembers.txt	Causes Haplotag to read a file containing consensus sequences from pre-selected (legacy) loci. After reading the file, Haplotag tries to combine the consensus tags into new clusters based on current parameters. Unlike "ClusterMergedAll", tags that remain as singletons will be kept in the cluster file because these may match multiple haplotypes in the next step.
!ReadMergedTags<br><br>*(multithreaded)*	MergedAll.txt	HTHaplos.txt	This procedure is designed to read a global tag count file, but it is only used when Haplotag has created clusters from predefined "legacy" loci (i.e. following ClusterGBSLoci). Haplotag then matches the tags to the cluster consensus sequences. All tags that can be matched are written to the file "HTHaplos.txt" for use in later steps.
!ReadClusters	HTClusters.txt	(none)	This step reads a set of cluster consensus sequences written in a previous step. Although clusters may remain in memory, this procedure needs to be executed in order to re-index the clusters and coordinate them with the haplotypes.
!ReadCMembers	HTMembers.txt	(none)	This step is optional. The file HTMembers is created when ClusterGBSLoci is run. Its purpose is to identify the names of legacy loci that may have been aligned into clusters, so they can be identified downstream in the new locus nomenclature.
!ReadHaplotypes	HTHaplos.txt	(none)	This step reads a set of haplotypes that have been indexed to a set of tag clusters.

			This file should be read immediately after reading HTClusters so that the haplotypes are properly indexed in memory. **Important: HTClusters and HTHaplos are indexed together and cannot be mixed or matched between projects.**
!MakeTagByTaxa<br><br>*(multithreaded)*	Path to directory of tag count files	HTBT.txt	This step requires that HTHaplos have been previously read. It will then search for these haplotypes in a set of tag count files, one per taxon, that have been produced by the UNEAK pipeline. As it does so, it builds a large matrix of tag counts for each combination of taxon (columns) and tags (rows).
!ReadFastQ<br><br>*(will soon be multithreaded)*	Path to directory of raw data files	HTBT.txt	This step does the same thing as MakeTagByTaxa, except that it parses reads for each taxa directly from raw FastQ files. This is intended to accelerate and simplify the calling of haplotypes from predefined models because the UNEAK pipeline is no longer required. In theory this step could be used in a discovery pipeline, but there is little point since UNEAK is still required to make a global tag count file from which to build clusters and/or efficiently identify haplotypes.
!ReadTBT	HTBT.txt		This reads a tag-by-taxa (TBT) file. It is not required if the TBT data are already in memory from a previous step (either MakeTagByTaxa or ReadFastQ). You may read the TBT file to re-start a previous analysis. E.g. to run model discovery (IdentifyAlleles) with different parameters. **It is absolutely essential that the TBT file matches HTClusters, HTHaplos and the HTTaxa from which it was built: do not change these files and do not mix projects**
!IdentifyAlleles	All required input needs to be in memory at this point.	HTLoci.txt<br>HTAlleles.txt<br>HTSNPs<br>HTGenos<br>HTSNPGenos<br>HTindex.htm<br>+ HTML passports	This is the final step in all pipelines. It behaves differently depending on whether the pipeline is in production mode or discovery mode. These differences are elaborated under "**Discovery vs. Production modes"** in the text. The primary result of this procedure is a set of genotype scores in either haplotype (HTGenos) or SNP (HTSNPGenos) format. This is also when the HTML passports are written. In discovery modes, new models are tested and selected and the files HTLoci and HTAlleles are written, while in production mode these files are used to define fixed models.

Discovery vs. Production Modes

Whether Haplotag runs in discovery mode vs production mode is determined by which files have been read at the time when !IdentifyAlleles is executed. If HTClusters and HTHaplos have been read, then it runs in a discovery mode. If HTLoci and HTAlleles have been read then it runs in production mode.

In discovery mode, Haplotag will start from a set of haplotypes that are assigned to clusters. For each cluster it will try to fit one or more loci that meets threshold filtering parameters. The best locus, which meets all filtering thresholds and fits the largest number of genotypes, will be selected first. Haplotypes that do not belong to that model may be assigned to subsequent models that also meet thresholds. In hexaploid oat, we often observe two good locus models from one cluster, sometimes three, and occasionally more. Each model will be given a locus identifier based on the cluster name. Haplotype- and SNP-based genotypes will be scored for each taxon based on presence (1 or more tags) or absence (no tags) of haplotypes from a given locus. The locus models, and genotypes for those models, will be reported in passport files, one passport per cluster. Locus models are combined by cluster because they most likely originate from duplicated genomic regions, which will be of interest to most genomic investigators. The cluster-based passport provides an intuitive way to visualize which bases are diagnostic among loci, and even which haplotype is ancestral. Also, by co-visualizing locus models built from the same cluster, aberrant behaviour requiring parameter optimization may be observed.

Discovery mode should be run across a large and diverse set of taxa, ideally containing a mix of progeny from diversity studies and from bi-parental populations. Diversity studies allow sampling of a large number of haplotypes, while bi-parental populations allow better validation that haplotypes are allelic. For example, segregation of haplotypes A *vs* B in one population and B vs C in another provides strong evidence that the locus model with three haplotypes is correct. Most taxa should be nearly homozygous so that a heterozygote frequency of approximately 10 or less can be applied to exclude spurious models that contain non-allelic haplotypes. However, if the majority of taxa are inbred, then it is possible to include sub-studies with heterozygous individuals. We are currently experimenting with the use of taxa belonging to different species and this may be reported in a future manuscript.

In production mode, it is assumed that you have tested and examined a good set of models in a diverse population where most haplotypes have already been incorporated into a model of Haplotag loci. Haplotag will then look for these exact same haplotypes in a new set of data, presumably from new taxa where genotypes are unknown. The demo files for production mode contain the same progeny as those for discovery modes only for validation purposes. An obvious criticism of production mode is that new haplotypes that were not seen previously in discovery mode will not be scored, and the data will appear as missing. Likewise, entirely new loci will also be ignored. The same criticism would be valid for any array-based SNP assay, which also detects only validated alleles at discovered loci. This is why it is critical to build models across diverse germplasm before using production mode. The primary advantage of production mode is that it allows fast and simple scoring of multiple new projects with an inter-compatible nomenclature. This may be important in collaborative studies that depend on an integrated genotype database. Another advantage of production mode is that it can be used with heterozygous progeny. In future we may develop routines that would allow the addition of new haplotypes and new locus models into an existing nomenclature.

Data files and output files

Depending on what pipeline functions are used, Haplotag will require or produce various input/output data. All types of data files are described in the reference table below, with detailed formatting requirements shown in the accompanying demo files. Those that are categorized as "input data" are used only for input (e.g. raw data or partially analysed data from other software), "output files" are produced by Haplotag, and those categorized as "output/input" are written by Haplotag for use in other Haplotag procedures. All files belonging to Haplotag (i.e. files other than those produced from other programs) have a header where the first two lines identify the type of file (e.g. "!Haplotag_FileType_HTKey") and the file version (e.g. !Haplotag_File_Version_3). These headers must be exactly as specified because Haplotag uses these in error checking. If changes are made to Haplotag that require a new file format, this will be indicated by a new file version. After the file type and file version, the header may contain an indefinite number of comments. The end of the comments and beginning of the data are signalled by a line containing only the text "!begin". After this, the format shown in the examples must be followed accurately. Unless specified otherwise, all terms (fields) must be separated by the TAB character.

Table 3. Description of all data files required by and/or produced by Haplotag. Simulated examples of each file (under the default filenames) are distributed with Haplotag. Detailed formatting requirements are provided in these examples.

Default Filename (refer to demo files)	Input, Output, or Both	Description
HTTaxa.txt	Input	This file contains a list of taxa (lines or accessions), one taxon per line. For each taxon, there is a short taxon ID (text or numeric) followed by a longer name, separated by a tab. Optionally, these may be followed by a third column that identifies a project name for each taxon. The order in which these taxa are listed will be the same order that they will appear in all output reports. In older versions, the first line of data gave the path to Tag count files; this is now optional and ignored.
HTKey.txt	Input	This file is currently required only for the production pipeline that reads raw FastQ files. It specifies, for each taxon, the raw data file and the unique barcode that is required to deconvolute the short reads. The raw data file is identified by the first two fields (Flowcell and Lane) which will be combined by Haplotag to form a file name in the format "Flowcell_lane_fastq.txt.gz". The last two fields identify the barcode, followed by the short taxa identifier. Taxa identifiers must match those in the HTTaxa file. If they are not present in HTTaxa they will be ignored and dropped from analysis.
Mergedall.txt	Input	This file is produced from the UNEAK pipeline (see **Short tutorial on running UNEAK**). It contains a list of all tags meeting a minimum presence threshold (default 10) across an entire project. Each tag is reported only once, followed by its length and a frequency count. This compresses a ginormous amount of information from the extremely large raw sequence files, and discards all tags that occur at very low frequency. By default, the MergedAll file from UNEAK is in a binary format that only UNEAK can read, but it can easily be extracted to a text file following instructions in the UNEAK tutorial.
Merged Tag counts (multiple files, see directory 'tagcounts'	Input	As above, this is a set of files produced by UNEAK. They are identical to the MergedAll file except that there is one for each taxa. They also need to be decoded from binary, and since UNEAK gives them

		unpredictably cryptic names, they must also be renamed to a short file name that matches the IDs in your taxa file. The renaming and decompression can be done by writing a batch file as discussed in the UNEAK tutorial.
Raw compressed FastQ file(s)	Input	FastQ files are the default data format for most short-read sequencers. These files will most likely be compressed into "gz" format. If not, you need to compress them using a gzip utility. The free software 7-Ziip can produce this format. Support for "zip" compression and uncompressed fastQ files in Haplotag may be added at a future date. The raw data files must be named with the format "Flowcell_lane_fastq.txt.gz", where Flowcell and Lane are identified in the HTKey file (this is an identical requirement in UNEAK).
GBSLoci.txt	Input	This input file format is provided for those who need to condense a set of previously identified GBS SNPs with reference sequences. Each tab-delimited record contains a reference name followed by a 64-base reference sequence followed by SNP alleles (A/C format) followed by the sequence length followed by the SNP position. The file does not contain a header, but may begin with comments that start with "#".
HTClusters.txt	Output/Input	This file is produced by either of the two clustering procedures (ClusterMergedAll or ClusterGBSLoci) and then it becomes the input for further pipeline steps. It has a header followed by a simple tab-delimited record format containing sequential cluster identifier, cluster name, reference sequence.
HTMembers.txt	Output/Input	This file is produced by the procedure ClusterGBSLoci. It lists all legacy loci that have been aligned into clusters. It can be read by later steps so that these legacy loci are incorporated into a new nomenclature.
HTHaplos.txt	Output/Input	HTHaplos is produced directly by ClusterMergedAll, or else it is produced by ReadMergedTags when the tags from MergedAll are aligned to the clusters from ClusterGBSLoci. HTHaplos defines all the unique tags that belong to each cluster, and which will be used to discover the haplotype models for that cluster.
HTClusterinfo.txt	Output/Input	This file is produced by the clustering procedures. It contains clusters and number of members before dropping clusters with more than @MaxTagsToTest, and renumbering clusters. It is not currently used in further analyses.
HTSingletons.txt	Output	This file is written to list the singletons from the ClusterMergedAll procedure. These singletons are not currently used in further Haplotag analysis.
HTBT.txt	Output/Input	The HTBT is a complete matrix of tag (haplotype) counts for all tags in the HTHaplos file (rows) by all taxa from the HTTaxa file (columns). The file contains a header, followed by two lines showing the number of tags and number of taxa. A tab delimited matrix (tags x taxa) follows. This file may be too large to open in a text editor, but there should be no need to edit it.
HTLoci.txt	Output/Input	This file contains a record of details for each locus defined by a selected haplotype model. It contains numerous details related to the model in which it was selected. It is written by IdentifyAlleles when running in discovery mode. This file is used as input for the production pipeline such that loci in different projects can have

		consistent naming conventions.
HTAlleles.txt	Output/Input	This file contains a record of details for each haplotype, indexed to a locus in the HTLocus file. It contains numerous details related to the model in which it was selected. It is written by IdentifyAlleles when running in discovery mode. This file is used as input for the production pipeline such that loci in different projects can have consistent naming conventions.
HTSNPs.txt	Output	This file is similar to HTHaplos except each record is for an individual SNP that is referenced to a specific position in the consensus sequence of a locus model.
HTGenos.txt	Output	This file contains the complete set of genotype scores for the population that was analysed. Genotypes are written in a haplotype format defined by the single-letter haplotype codes assigned in the HTHaplos file. In production mode, genotypes may be completely missing from some loci, so these are not written even though they may appear in the Passport files.
HTSNPGenos.txt	Output	This file is similar to HTGenos file except that each record is for the genotypes of an individual SNP that is referenced to the HTSNPs file.
HTindex.html	Output	This is a single HTML file that can be opened in any web browser, probably by just clicking on it. This provides a searchable index of all clusters for which there is at least one selected locus model. The links within HTindex.html go directly to the corresponding passport for that cluster. For a very large project the HTindex may load slowly. For this reason we recommend using the option "@ThreePlus true". This will index only the clusters where a locus has more than two haplotypes, which are the most interesting to look at. Other clusters are still written to passports so you can find them by typing their name in the browser address bar.
HTPassports (multiple files)	Output	Each cluster with at least one validated locus model will have an HTML passport. The passports follow the format shown in Figure 1. The first alignment always shows the complete cluster and its consensus. After that there will be alignments and consensus sequences for each selected model. After that there will be a table of haplotype counts, arranged and coloured by the locus model to which they belong. In discovery mode, haplotypes that do not fit a locus model are shown last and coloured grey. The passports are all written inside a subdirectory within your primary output directory. We do not recommend browsing this directory if your project has a large number of clusters; instead you can use the HTindex file (above) to locate a cluster of interest.

Standard Haplotag Pipelines

Haplotag was designed to work from three different starting points, as illustrated in **Figure 2**, all resulting in the same type of output. Our reasons for splitting these pipelines into component steps were (1) to modularize the pipelines, clarify how they work, show which steps are shared, and allow potential modifications to be made, and (2) so that a failed pipeline might be re-started midway through an analysis, or later steps (e.g. model fitting with different parameters) could be re-tested more efficiently from intermediate files.

The easiest way to get started with Haplotag is to unpack the distribution archive, then look for three demo directories named according to the pipelines described in **Table 4** and illustrated in **Figure 2**. Each directory contains a fully functional Haplotag script that works with a set of small annotated demo files. The script will analyse a simulated GBS project using only the data files contained in that directory. These data files are deliberately small and idealized so that the pipeline will run quickly and produce informative output. Each demo directory will also contain a batch file to start the program. If you maintain the directory unpacked from the zip file on a Windows machine, these batch files called "run_demo.bat" can be double-clicked to run the included demo (although you should never click on files like this until you inspect what they are about to do, even if you trust the author ;-). After running, the output should appear in a new subdirectory called "output". There will already be a subdirectory directory called "expected_output". The contents of these two directories should be identical, unless you experiment with the input files to see what happens (highly recommended).

Table 4. Standard Haplotag pipelines. See **Figure 2** for illustration. See also the demo files which are designed to work with the sample data files provided with Haplotag.

**ID Fig. 2**	**Sample input directory**	**Description of application**	**Pipeline Steps (Table 2)**	**Input required (Table 3)**
Disc1	Demo_Discover _from_UNEAK	Discover, test, and report GBS haplotype models and genotypes starting with merged tag counts from UNEAK pipeline.	!ReadTaxaIDFile<br>!ClusterMergedAll<br>!ReadClusters<br>!ReadHaplotypes<br>!MakeTagByTaxa<br>!IdentifyAlleles	HTTaxa.txt<br>MergedAll.txt<br><br>+ tag counts from UNEAK
Disc2	Demo_Discover _from_GBSLoci	Discover, test, and report GBS haplotype models and genotypes starting with reference tags from previous GBS analyses, preserving legacy nomenclature. It should also be possible to use predicted fragments from a reference genome as source tags in GBSLoci.txt.[3]	!ReadTaxaIDFile<br>!ClusterGBSLoci<br>!ReadClusters<br>!ReadCMembers<br>!ReadMergedTags<br>!ReadHaplotypes<br>!MakeTagByTaxa<br>!IdentifyAlleles	HTTaxa.txt<br>GBSLoci.txt<br>MergedAll.txt<br><br>+ tag counts from UNEAK
Prod1	Demo_Producti on_From_UNEA K	Analyse and report previously discovered GBS haplotype models based on new data and new taxa, where tag counts for each taxon have been made using UNEAK. Models are defined by HTLoci and HTAlleles produced from a discovery run.	!ReadTaxaIDFile<br>!ReadLoci<br>!ReadAlleles<br>!MakeTagByTaxa<br>!IdentifyAlleles	HTTaxa.txt<br><br>HTLoci.txt<br>HTAlleles.txt<br><br>+ tag counts from UNEAK
Prod2	Demo_Producti on_from_FASTQ	Analyse and report previously discovered GBS haplotype models based on new data and new taxa, where sequences are read directly from compressed FastQ files.	!ReadTaxaIDFile<br>!ReadTaxaKey<br>!ReadLoci<br>!ReadAlleles	HTTaxa.txt<br>HTKey.txt<br>HTLoci.txt<br>HTAlleles.txt

[3] We have not tested this, but it may be possible to compile a set of predicted reference tags from a reference genome into the file 'GBSLoci.txt", such that Haplotag will build similarity clusters with preserved reference names (written to HTMembers). Haplotag would then build locus models from within these clusters based on the matched haplotypes, thus validating and cross-referencing sets of paralogous loci.

		Models are defined by HTLoci and HTAlleles produced from a discovery run.	!ReadFastQ<br>!IdentifyAlleles	Raw FastQ files

Short tutorial on running UNEAK.

When we developed Haplotag we were already using UNEAK, which is an excellent GBS pipeline that produces robust GBS data. We recognized that UNEAK was very efficient at reducing raw data files to lists of tags with counts of their occurrence, and that this was a prerequisite for any further analysis. We chose not to duplicate this functionality, although we plan to address this in future because UNEAK is no longer being developed and may never handle longer sequence reads. Currently, to use Haplotag in any mode you will need to run the first steps of UNEAK to produce global tag counts (Mergedall.txt) and to produce a separate tag count file for each taxon that you wish to analyse in Haplotag.

The UNEAK pipeline is part of the TASSEL-3 distribution and is not available in other TASSEL versions. Please locate TASSEL-3 and the UNEAK user manual which are now located in the TASSEL "archived" directory: http://www.maizegenetics.net/#!tassel/. The purpose of this section is to provide a few additional pointers on using UNEAK to supplement the manual. Examples and scripts here are provided for the Windows environment, since this environment is less well supported in the TASSEL manual.

Unless you are handling a relatively small data set, you will likely need to install a JAVA 64-bit environment. The default JAVA environment is 32-bit even on a 64-bit machine. You may install both JAVA environments on one machine.

The next thing to check after downloading and installing TASSEL is to prepare a master-script that will be called from your UNEAK project folder. It is best to keep this in the TASSEL install directory and point to it from a separate batch file within your project directory. Windows will use a file such as "run_pipeline.bat" while Linux will use the PERL script (run_pipeline.pl).

We recommend that you make a copy of the run_pipeline.bat file and name it something like "run_pipeline_HiMem.bat". Then edit the file as shown below (comments are followed by double colons):

```
@echo off
:: change TOP= to the location where TASSEL is installed:
set TOP=C:\TASSEL3
set LIB_JARS=%TOP%\lib
set CP=%TOP%\sTASSEL.jar
for %%i in (%LIB_JARS%\*.jar) do call "%TOP%\cp.bat" %%i
echo %CP%
:: in the statement below, 20G and 40G are the starting and maximum memory sizes in GB
:: Make sure that -Xmx is set to a size that can safely run on your computer
java -classpath "%CP%" -Xms20G -Xmx40G net.maizegenetics.pipeline.TasselPipeline %*
```

You should set up a separate directory for each project where you will use UNEAK. For example, you if you have a data directory on drive D this could be "D:\data\GBS\UNEAK\myproject1". Inside this directory you should prepare a text file with a .bat extension. The example below has comments for explanation, and is designed to pause after creating a directory hierarchy so that you can put the correct input files into the new subdirectories that it will create.

```
:: set the next line to point to your master TASSEL batch file:
SET PROG=C:\TASSEL3\run_pipeline_himem.bat
:: The next line sets the default directory from which you run this batch program
SET DIR=%~dp0
:: The next line will create the required UNEAK directory structure for you:
call %PROG% -fork1 -UCreatWorkingDirPlugin -w %DIR% -endPlugin -runfork1
```

```
echo  The program will now pause while you put correct input files into the new directories
echo   -  put your key file into the "key" directory
echo   -  put your raw sequence files into the "illumina" directory
pause
:: Make sure to edit the TASSEL parameters below such that they are appropriate for your work:
call %PROG% -fork1 -UFastqToTagCountPlugin -w %DIR% -e PstI-MspI -endPlugin -runfork1
call %PROG% -fork1 -UMergeTaxaTagCountPlugin -w %DIR%  -m 250000000 -c 50 -endPlugin -runfork1
:: The next line will make a text version of mergedall
call %PROG% -fork1 -BinaryToTextPlugin -i %DIR%\mergedTagCounts\mergedAll.cnt^
  -o %DIR%\mergedTagCounts\mergedAll.txt -t TagCounts -endPlugin -runfork1
:: The remaining UNEAK pipeline can be run for comparison
:: or you can stop here and run Haplotag.
```

After running UNEAK, you will need to convert the tag count files from binary format (extension ".cnt") to text format (extension ".txt"). The Mergedall file is converted in the above script, but you will need to mess around a bit to convert all the individual taxa count files that are found within the TagCounts directory. An example is below:

```
:: Batch file for conversion of binary UNEAK files to text
:: This is a comment line
:: First I like to set a variable that points to the directory where TASSEL is stored
SET PROG=S:\TASSEL\TASSEL3\run_pipeline_HiMem.bat
:: To convert tag count file for each taxa:
call %PROG% -fork1 -BinaryToTextPlugin -i T1587.cnt -o T1587.txt -t TagCounts -endPlugin -runfork1
call %PROG% -fork1 -BinaryToTextPlugin -i T1588.cnt -o T1588.txt -t TagCounts -endPlugin -runfork1
call %PROG% -fork1 -BinaryToTextPlugin -i T1589.cnt -o T1589.txt -t TagCounts -endPlugin -runfork1
```

If taxa.cnt files are numbered sequentially with a consistent pattern of filenames it is possible to write a batch file (or a script if you are running Linux) that will loop through these names automatically. An example of this is below.

```
:: script to convert 1248 TEXT COUNT files named sequentially

SET PROG=S:\NICK_SHARED\bin\TASSEL\TASSEL3\run_pipeline_himem.bat
SET start=1
SET last=1248


set /a i=%start%

:LOOP_BEGIN
IF %i% GTR %last% GOTO END
echo This is iteration %i%.
SET TAX=P%i%
call %PROG% -fork1 -BinaryToTextPlugin -i .\tagcounts_a\%TAX%_merged.cnt -o
.\txtcounts\%TAX%.txt -t TagCounts -endPlugin -runfork1
SET /a i=%i%+1
GOTO LOOP_BEGIN
:END

pause
```

Updates and bug fixes: (by date of released version)

- 2016-January-10
  - Added a feature to specify a threshold for allele ratio when calling haplotypes. This is controlled in the input file using @HetRatio followed by a decimal between zero and 1, which specifies a threshold ratio of tag count for minor allele, below which the allele will be dropped.
- 2015-April-30
  - Corrected MinTagCount threshold to keep >= this number of tags from MergedAll (previously it used only greater than).
  - Added space between TagID and Count in passport files so these numbers won't run together. Also made a footnote that Count means number of taxa with a given haplotype.
- 2015-April-07
  - Fixed bug in ClusterMergedAll that crashed with a small number of tags and a large number of processors.
- 2015-April-08
  - Extended the haplotype character naming convention to simulate multi-allele data for use in TASSEL, following convention given here: https://bitbucket.org/tasseladmin/tassel-5-source/wiki/UserManual/Load/DataFAQ
  - A SNP nomenclature file (HTSNPs.txt) and a SNP genotype file (HTSNPGenos) are now created alongside of the haplotype-based output, for those preferring to perform SNP-based genotype analysis.

ToDo: (Planned fixes, short-term)

- Add reporting of legacy SNP names to the SNP nomenclature file (applies only to discovery mode from a GBSLoci file) where previous SNP names may exist.
- Add additional columns to verbose model selection reports to give allele frequencies etc. (it is not always clear why a model is rejected)
- Double check and document mintagcount meaning. Note that currently it affects both clustering from mergedall as well as clusters from GBSloci (so setting >1 eliminates everything loci from GBSloci). It should only affect Mergedall. Change examples to 10 so that people set these higher by default!
- Fix CbyT to read and report sequences – especially so that it works to update GBSLoci nomenclature file.
- When reading tag count files in the "!MakeTagByTaxa" step, if a taxa file is listed in the HTTaxa input list but the file is not found, the thread that is reading that taxa file will stall with no error message, or if there is an error message it will be lost in the long string of progress reports. Then the program will stop, and the user will not know why. There needs to be an alert and graceful exit. Better still, there should be a preprocessing step to check for the presence of all files before this step is executed.
- There is a bug in hapltag discovery mode. When GBSLoci are clustered, if no clusters are formed (i.e. every tag is unique), the program will crash.

Warnings, known issues, and limitations

- Haplotag numbers SNP positions starting with 1 because this is more intuitive for most biologists. However other pipelines (e.g. TASSEL and UNEAK) identify positions starting with zero (so the last base in a 64-base tag will be 63). This is because most computer languages work this way. Please be careful when cross-referencing output from Haplotag and other pipelines.
- You cannot use spaces in file names or file paths. Microsoft should never have allowed this in the first place. If you have your data files in a location such as “c:\users\me\my documents\” then we suggest you move your data to a new directory such as “d:\data\Haplotag\project1”.
- Haplotag does not perform checks of available RAM. It works with dynamic arrays and it is difficult to predict in advance how large these arrays will grow. If you are working with very large files and limited RAM, it is a good idea to watch your physical RAM usage in Task manager. The program (and your computer) will run very slowly if your RAM fills up to near maximum.
- Haplotag currently works only with 64 base sequences (usually trimmed from 100-base single-end reads) because it is designed to be inter-compatible with the UNEAK pipeline which has the same limitation. We may extend this capability in future.
- The feature to read raw compressed FASTQ files will only work with Illumina-type output with fixed-length reads equal to or greater than 100 bases. Haplotag will not read Ion Proton files because these have variable length reads that are often shorter than 64 bases. If there is demand, we may produce a utility to convert these files, padding the lengths to 100 bases.
- The feature to read FASTQ files is only designed to work in production mode with a set of predefined haplotypes. Currently, the discovery mode requires that raw reads be first processed into tag counts using the UNEAK pipeline. The UNEAK/TASSEL algorithms are very efficient at this step, so we may or may not replicate this feature in future.
