# Supplementary material for: Haplotag: Software for Haplotype-Based Genotyping-by-Sequencing Analysis
Source: G3 (Bethesda). 2016 Jan 26;6(4):857–63. doi: 10.1534/g3.115.024596 (PMC4825656; doi:10.1534/g3.115.024596)
Supplement: Supporting Information [file supp_g3.115.024596_FileS2.zip › S2 Model Selection Details.htm]

  Details of model selection for:   HC1   
  Note: verbose mode was set to ON .....
 I am reporting all 57 possible locus models (i.e. all combinations of haplotype tags) for this tag cluster
  "Offender" identifies the haplotype in a model that introduces the maximum hetozygote frequency relative to its presence
  "Acceptable" means that a model meets minimum filtering thresholds.  Models that fit the most genotypes are selected from these.
 
  Model  Member alleles   Genos   Geno%   Het%   Trihet%   Multihet%   MaxHF%   Offender   Acceptable
  &nbsp1 &nbsp 1 2 3 4 5 6 &nbsp10   100.0&nbsp 30.0&nbsp  70.0&nbsp 0.0&nbsp 300.0&nbsp &nbsp&nbsp3 
  &nbsp2 &nbsp 1 2 3 4 5 &nbsp10   100.0&nbsp 40.0&nbsp  50.0&nbsp 0.0&nbsp 100.0&nbsp &nbsp&nbsp2 
  &nbsp3 &nbsp 1 2 3 4 6 &nbsp10   100.0&nbsp 70.0&nbsp  30.0&nbsp 0.0&nbsp 300.0&nbsp &nbsp&nbsp3 
  &nbsp4 &nbsp 1 2 3 5 6 &nbsp10   100.0&nbsp 50.0&nbsp  50.0&nbsp 0.0&nbsp 300.0&nbsp &nbsp&nbsp3 
  &nbsp5 &nbsp 1 2 4 5 6 &nbsp10   100.0&nbsp 40.0&nbsp  60.0&nbsp 0.0&nbsp 125.0&nbsp &nbsp&nbsp6 
  &nbsp6 &nbsp 1 3 4 5 6 &nbsp10   100.0&nbsp 90.0&nbsp  10.0&nbsp 0.0&nbsp 300.0&nbsp &nbsp&nbsp3 
  &nbsp7 &nbsp 2 3 4 5 6 &nbsp10   100.0&nbsp 90.0&nbsp  10.0&nbsp 0.0&nbsp 100.0&nbsp &nbsp&nbsp2 
  &nbsp8 &nbsp 1 2 3 4 &nbsp10   100.0&nbsp 60.0&nbsp  20.0&nbsp 0.0&nbsp 100.0&nbsp &nbsp&nbsp3 
  &nbsp9 &nbsp 1 2 3 5 &nbsp10   100.0&nbsp 60.0&nbsp  30.0&nbsp 0.0&nbsp 100.0&nbsp &nbsp&nbsp2 
  &nbsp10 &nbsp 1 2 3 6 &nbsp10   100.0&nbsp 90.0&nbsp  10.0&nbsp 0.0&nbsp 300.0&nbsp &nbsp&nbsp3 
  &nbsp11 &nbsp 1 2 4 5 &nbsp10   100.0&nbsp 40.0&nbsp  40.0&nbsp 0.0&nbsp 100.0&nbsp &nbsp&nbsp2 
  &nbsp12 &nbsp 1 2 4 6 &nbsp10   100.0&nbsp 60.0&nbsp  30.0&nbsp 0.0&nbsp 125.0&nbsp &nbsp&nbsp6 
  &nbsp13 &nbsp 1 2 5 6 &nbsp10   100.0&nbsp 60.0&nbsp  40.0&nbsp 0.0&nbsp 125.0&nbsp &nbsp&nbsp6 
  &nbsp14 &nbsp 1 3 4 5 &nbsp10   100.0&nbsp 50.0&nbsp  10.0&nbsp 0.0&nbsp 100.0&nbsp &nbsp&nbsp3 
  &nbsp15 &nbsp 1 3 4 6 &nbsp10   100.0&nbsp 70.0&nbsp  0.0&nbsp 0.0&nbsp 300.0&nbsp &nbsp&nbsp3 
  &nbsp16 &nbsp 1 3 5 6 &nbsp10   100.0&nbsp 70.0&nbsp  10.0&nbsp 0.0&nbsp 300.0&nbsp &nbsp&nbsp3 
  &nbsp17 &nbsp 1 4 5 6 &nbsp10   100.0&nbsp 100.0&nbsp  0.0&nbsp 0.0&nbsp 100.0&nbsp &nbsp&nbsp1 
  &nbsp18 &nbsp 2 3 4 5 &nbsp10   100.0&nbsp 60.0&nbsp  0.0&nbsp 0.0&nbsp 100.0&nbsp &nbsp&nbsp4 
  &nbsp19 &nbsp 2 3 4 6 &nbsp10   100.0&nbsp 70.0&nbsp  0.0&nbsp 0.0&nbsp 100.0&nbsp &nbsp&nbsp4 
  &nbsp20 &nbsp 2 3 5 6 &nbsp10   100.0&nbsp 70.0&nbsp  10.0&nbsp 0.0&nbsp 100.0&nbsp &nbsp&nbsp3 
  &nbsp21 &nbsp 2 4 5 6 &nbsp10   100.0&nbsp 60.0&nbsp  10.0&nbsp 0.0&nbsp 100.0&nbsp &nbsp&nbsp2 
  &nbsp22 &nbsp 3 4 5 6 &nbsp10   100.0&nbsp 40.0&nbsp  0.0&nbsp 0.0&nbsp 100.0&nbsp &nbsp&nbsp3 
  &nbsp23 &nbsp 1 2 3 &nbsp10   100.0&nbsp 80.0&nbsp  0.0&nbsp 0.0&nbsp 100.0&nbsp &nbsp&nbsp3 
  &nbsp24 &nbsp 1 2 4 &nbsp10   100.0&nbsp 40.0&nbsp  20.0&nbsp 0.0&nbsp 100.0&nbsp &nbsp&nbsp4 
  &nbsp25 &nbsp 1 2 5 &nbsp10   100.0&nbsp 60.0&nbsp  20.0&nbsp 0.0&nbsp 100.0&nbsp &nbsp&nbsp2 
  &nbsp26 &nbsp 1 2 6 &nbsp10   100.0&nbsp 80.0&nbsp  10.0&nbsp 0.0&nbsp 125.0&nbsp &nbsp&nbsp6 
  &nbsp27 &nbsp 1 3 4 &nbsp9   90.0&nbsp 44.4&nbsp  0.0&nbsp 0.0&nbsp 100.0&nbsp &nbsp&nbsp3 
  &nbsp28 &nbsp 1 3 5 &nbsp10   100.0&nbsp 30.0&nbsp  10.0&nbsp 0.0&nbsp 100.0&nbsp &nbsp&nbsp3 
  &nbsp29 &nbsp 1 3 6 &nbsp10   100.0&nbsp 50.0&nbsp  0.0&nbsp 0.0&nbsp 300.0&nbsp &nbsp&nbsp3 
  &nbsp30 &nbsp 1 4 5 &nbsp10   100.0&nbsp 50.0&nbsp  0.0&nbsp 0.0&nbsp 100.0&nbsp &nbsp&nbsp4 
  &nbsp31 &nbsp 1 4 6 &nbsp10   100.0&nbsp 60.0&nbsp  0.0&nbsp 0.0&nbsp 100.0&nbsp &nbsp&nbsp4 
  &nbsp32 &nbsp 1 5 6 &nbsp10   100.0&nbsp 80.0&nbsp  0.0&nbsp 0.0&nbsp 100.0&nbsp &nbsp&nbsp5 
  &nbsp33 &nbsp 2 3 4 &nbsp10   100.0&nbsp 20.0&nbsp  0.0&nbsp 0.0&nbsp 100.0&nbsp &nbsp&nbsp4 
  &nbsp34 &nbsp 2 3 5 &nbsp10   100.0&nbsp 40.0&nbsp  0.0&nbsp 0.0&nbsp 100.0&nbsp &nbsp&nbsp5 
  &nbsp35 &nbsp 2 3 6 &nbsp10   100.0&nbsp 50.0&nbsp  0.0&nbsp 0.0&nbsp 100.0&nbsp &nbsp&nbsp6 
  &nbsp36 &nbsp 2 4 5 &nbsp8   80.0&nbsp 62.5&nbsp  0.0&nbsp 0.0&nbsp 100.0&nbsp &nbsp&nbsp4 
  &nbsp37 &nbsp 2 4 6 &nbsp9   90.0&nbsp 55.6&nbsp  0.0&nbsp 0.0&nbsp 100.0&nbsp &nbsp&nbsp4 
  &nbsp38 &nbsp 2 5 6 &nbsp10   100.0&nbsp 40.0&nbsp  10.0&nbsp 0.0&nbsp 75.0&nbsp &nbsp&nbsp5 
  &nbsp39 &nbsp 3 4 5 &nbsp8   80.0&nbsp 12.5&nbsp  0.0&nbsp 0.0&nbsp 33.3&nbsp &nbsp&nbsp3 
  &nbsp40 &nbsp 3 4 6 &nbsp8   80.0&nbsp 25.0&nbsp  0.0&nbsp 0.0&nbsp 66.7&nbsp &nbsp&nbsp3 
  &nbsp41 &nbsp 3 5 6 &nbsp8   80.0&nbsp 50.0&nbsp  0.0&nbsp 0.0&nbsp 100.0&nbsp &nbsp&nbsp3 
  &nbsp42 &nbsp 4 5 6 &nbsp10   100.0&nbsp 10.0&nbsp  0.0&nbsp 0.0&nbsp 25.0&nbsp &nbsp&nbsp5  YES
  &nbsp43 &nbsp 1 2 &nbsp10   100.0&nbsp 60.0&nbsp  0.0&nbsp 0.0&nbsp 85.7&nbsp &nbsp&nbsp2 
  &nbsp44 &nbsp 1 3 &nbsp9   90.0&nbsp 22.2&nbsp  0.0&nbsp 0.0&nbsp 100.0&nbsp &nbsp&nbsp3 
  &nbsp45 &nbsp 1 4 &nbsp9   90.0&nbsp 22.2&nbsp  0.0&nbsp 0.0&nbsp 100.0&nbsp &nbsp&nbsp4 
  &nbsp46 &nbsp 1 5 &nbsp10   100.0&nbsp 30.0&nbsp  0.0&nbsp 0.0&nbsp 75.0&nbsp &nbsp&nbsp5 
  &nbsp47 &nbsp 1 6 &nbsp10   100.0&nbsp 40.0&nbsp  0.0&nbsp 0.0&nbsp 80.0&nbsp &nbsp&nbsp6 
  &nbsp48 &nbsp 2 3 &nbsp10   100.0&nbsp 0.0&nbsp  0.0&nbsp 0.0&nbsp 0.0&nbsp &nbsp&nbsp0  YES
  &nbsp49 &nbsp 2 4 &nbsp7   70.0&nbsp 28.6&nbsp  0.0&nbsp 0.0&nbsp 100.0&nbsp &nbsp&nbsp4 
  &nbsp50 &nbsp 2 5 &nbsp8   80.0&nbsp 37.5&nbsp  0.0&nbsp 0.0&nbsp 75.0&nbsp &nbsp&nbsp5 
  &nbsp51 &nbsp 2 6 &nbsp9   90.0&nbsp 33.3&nbsp  0.0&nbsp 0.0&nbsp 60.0&nbsp &nbsp&nbsp6 
  &nbsp52 &nbsp 3 4 &nbsp5   50.0&nbsp 0.0&nbsp  0.0&nbsp 0.0&nbsp 0.0&nbsp &nbsp&nbsp0  YES
  &nbsp53 &nbsp 3 5 &nbsp6   60.0&nbsp 16.7&nbsp  0.0&nbsp 0.0&nbsp 33.3&nbsp &nbsp&nbsp3 
  &nbsp54 &nbsp 3 6 &nbsp6   60.0&nbsp 33.3&nbsp  0.0&nbsp 0.0&nbsp 66.7&nbsp &nbsp&nbsp3 
  &nbsp55 &nbsp 4 5 &nbsp6   60.0&nbsp 0.0&nbsp  0.0&nbsp 0.0&nbsp 0.0&nbsp &nbsp&nbsp0  YES
  &nbsp56 &nbsp 4 6 &nbsp7   70.0&nbsp 0.0&nbsp  0.0&nbsp 0.0&nbsp 0.0&nbsp &nbsp&nbsp0  YES
  &nbsp57 &nbsp 5 6 &nbsp8   80.0&nbsp 12.5&nbsp  0.0&nbsp 0.0&nbsp 25.0&nbsp &nbsp&nbsp5 
  
*Filtering thresholds are: 
   Completeness (minimum proportion of genotypes relative to total taxa number) >= 0.40
   Heterozygotes  Tri-Zygotes  Minimum Haplotype Frequency >=0.10; Maximum Haplotype Frequency <=0.99
